# Unilateral Vestibular Loss Impairs External Space Representation

**DOI:** 10.1371/journal.pone.0088576

**Published:** 2014-02-11

**Authors:** Liliane Borel, Christine Redon-Zouiteni, Pierre Cauvin, Michel Dumitrescu, Arnaud Devèze, Jacques Magnan, Patrick Péruch

**Affiliations:** 1 Aix-Marseille Université, Marseille, France; 2 CNRS, UMR 7260 Laboratoire de Neurosciences Intégratives et Adaptatives, Marseille, France; 3 Service d'Oto-Rhino-Laryngologie et Chirurgie Cervico-Faciale, Hôpital Nord, Marseille, France; 4 INSERM, UMR_S 1106 Institut de Neurosciences des Systèmes, Marseille, France; University of Alberta, Canada

## Abstract

The vestibular system is responsible for a wide range of postural and oculomotor functions and maintains an internal, updated representation of the position and movement of the head in space. In this study, we assessed whether unilateral vestibular loss affects external space representation. Patients with Menière's disease and healthy participants were instructed to point to memorized targets in near (peripersonal) and far (extrapersonal) spaces in the absence or presence of a visual background. These individuals were also required to estimate their body pointing direction. Menière's disease patients were tested before unilateral vestibular neurotomy and during the recovery period (one week and one month after the operation), and healthy participants were tested at similar times. Unilateral vestibular loss impaired the representation of both the external space and the body pointing direction: in the dark, the configuration of perceived targets was shifted toward the lesioned side and compressed toward the contralesioned hemifield, with higher pointing error in the near space. Performance varied according to the time elapsed after neurotomy: deficits were stronger during the early stages, while gradual compensation occurred subsequently. These findings provide the first demonstration of the critical role of vestibular signals in the representation of external space and of body pointing direction in the early stages after unilateral vestibular loss.

## Introduction

The vestibular system is involved in a wide range of functions, from postural and oculomotor reflexes to spatial cognition. Vestibular organs (semicircular canals and otoliths) measure head angular and linear accelerations, respectively, for the coding and representation of head movements in space. Projections to ocular and spinal motoneurons mediate oculomotor and postural reflexes involved in stabilizing and orienting the gaze, head and body in space [1], [2]. Vestibular signals are also important for self-perception through sensations associated with the whole-body location and motion in space and the displacement of the environment relative to the individual [3], [4].

Asymmetry in vestibular signals in patients with unilateral peripheral vestibular loss induces asymmetrical postural, locomotor, oculomotor and perceptive responses [5], [6]. This syndrome results in head and trunk tilts toward the lesioned side [7], spontaneous nystagmus, ocular torsion and gaze stabilization deficits associated with an asymmetric vestibulo-ocular reflex [8]. Patients with unilateral loss perceive the visual vertical as deviated toward the lesioned side [9]. Previous studies have shown that vestibular signals also participate in detecting and estimating body displacements, as demonstrated by changes in goal-directed locomotion (e.g. [10], [11]) and difficulties navigating the physical environment [12]–[14]. Moreover, vestibular signals maintain an internal, updated representation of body position and movement in space when only visual cues are available [13]–[15]. Furthermore, mental imagery studies have revealed a critical role for vestibular signals in the processing of metric properties of mental representations [16] and mental rotational abilities [16], [17].

The function of the vestibular system in spatial cognition is just being recognized, and the consequences of its loss at a higher level are important to acknowledge. The properties of the extrapersonal space have long been considered to be independent of vestibular system properties. In the present study, we determined whether symmetrical vestibular signals are necessary for the accurate representation of the external space. In healthy subjects, vestibular signals are used to estimate the target location in space during self-motion [18], [19]. Thus, patients with unilateral vestibular loss might experience the impaired representation of object location in space. Here, we investigate visual target location in Menière's disease patients who underwent unilateral vestibular neurotomy as a surgical treatment for the incapacitating form of peripheral vertigo encountered in this disease. The target location was recorded in the absence of subject motion, i.e. in the absence of vestibular stimulation.

Pointing to memorized targets involves two distinct mental spatial representations: egocentric (or body-centered) and allocentric (or environment-centered) (e.g. [20], [21]). In the present study, to discriminate between egocentric and allocentric spatial representations, pointing performance was recorded in the dark and in the light using a visually structured background to determine whether the ability to locate a target was impaired in darkness. Locating a target in the dark involves an egocentric reference frame, while the presence of visual background information in the light requires an allocentric reference frame that serves as a common reference for target and body location. The allocentric reference frame increases the pointing accuracy of patients and healthy subjects [22]. Here, we examined egocentric target representation in the objective median plane and in different ipsilateral and contralateral hemifield locations to define potential asymmetries in the external space representation. Changes in the target location representation were measured in the different Cartesian directions.

Other contextual factors, such as the distance between the participant and the target, might differentially influence the representation of the target location. Indeed, a distinction between near (peripersonal) and far (extrapersonal) space coding has been reported in brain-damaged patients [23], [24] with damage in the vestibular projection areas. Thus, our goal was to determine whether asymmetry in vestibular inputs is characterized by changes in spatial representation in the near and far spaces. To this end, participants were required to locate visual targets at variable distances relative to their midline projection.

Relatively few attempts have been made to quantify the consequences of vestibular loss in body cognition. Indeed, the body schema is modified after vestibular loss, resulting in an altered representation of the width of hemi-bodies [25], [26]. Moreover, it has been shown that vestibular signals are necessary for the accurate representation of body pointing direction, as shown by changes in the visual localization of subjective straight ahead (SSA), which indicates the location of the body mid-sagittal plane. Previous studies have revealed a SSA deviation in the acute [27], [28] and chronic stages several years after unilateral vestibular loss [29]. However, it remains unclear whether changes in the representation of the external world interact with the representation of body pointing direction. Thus, visual SSA deviation was associated with deviations in visual target location.

Previous studies have shown that spatial impairments are maximal during the early stages following vestibular asymmetry [3], [16], [30], [31]. Therefore, to specify the recovery time-course of spatial deficits, the performance of the patients was compared with that of healthy control participants before unilateral vestibular neurotomy (UVN), in the early stages after UVN (one week after) and during vestibular compensation (one month after). We hypothesized that both peripersonal and extrapersonal space representations are affected during the early stages of unilateral loss and that recovery occurs gradually over time. Moreover, we also expected that changes in representation of body pointing direction one week after UVN might lead to the inaccurate representation of the external world.

## Methods

### Participants

The experiments were performed in 13 unilateral vestibular-defective (UVD) patients suffering from Menière's disease (seven women, six men; mean age±SD: 49.2±11.2 years; Table 1). The patients exhibited the classic triadic syndrome of hearing loss, tinnitus and recurrent vertigo. The unilateral vestibular loss, determined through bithermal caloric irrigation with cold (30°C) and warm (44°C) water, averaged 26.6±10.9%, and the hearing loss averaged 45.2±23.8 dB in the affected ear. Patients had been affected for an average of 6.2±6.7 years. Because these patients are typically resistant to anti-vertigo drugs, curative unilateral vestibular neurotomy (UVN) was performed. The surgical procedure involved retrosigmoid vestibular neurotomy [32]. Because the side of vestibular loss might influence changes in cognitive processing [29], [33], all patients enrolled in the present study had a UVN on the right side. The performance of these patients was compared with that of 13 healthy participants (eight women, five men; mean age: 48.7±10 years) without a history of vestibular or oto-neurological diseases. All participants were right-handed with normal or corrected-to-normal vision. Each participant provided written informed consent, and this study was approved by the local Ethics Committee (CCP Marseille-Sud II # 07 010).

**Table 1:** Demographic and clinical data for the patients and controls.

Patients						Controls		
Gender	Age	History	Vestibular	Hearing	Education	Gender	Age	Education
	(years)	(years)	loss (%)	loss (dB)	(years)		(years)	(years)
M	47	6	9	58	9	F	60	14
F	37	7	20	29	17	F	57	9
M	47	1	28	27	10	F	51	8
F	64	9	22	Cophosis	8	F	41	14
M	27	4	24	60	14	M	62	22
F	58	2	51	62	10	M	41	22
F	61	7	29	63	12	M	57	15
M	59	1	17	52	10	M	45	14
M	43	5	34	37	17	F	28	18
F	45	6	35	14	18	F	50	14
F	56	27	-	38	18	F	49	14
M	59	5	-	93	10	M	56	22
F	37	1	24	10	15	F	37	21

"-" indicates data not available.

### Experimental sessions

The patients and controls were tested during three experimental sessions. The patients were tested before UVN (D−1), when they had not received antivertigo medications, and they did not report attacks of vertigo during the preceding week. The patients' performance was recorded at two postoperative times throughout the recovery process: one week after UVN (D+7), where all patients were able to stand straight and motionless, and one month after UVN (D+30). The intervals between the three experimental sessions were the same for the healthy participants.

### Target location

#### Experimental set-up

The participants were seated, and their head was immobilized on a head-and-chin rest. The heights of the seat and resting system were independently adjusted until the participants felt that they were in a comfortable position, with their head aligned with their trunk and their midline aligned with the center of the experimental space. The participants held a laser pointer with both hands, which rested on a small platform located at chest level. Pointing to the target location only required rotating the wrists while keeping the hands on the platform. Each subject wore home-made glasses that limited the visual field to approximately 60 degrees horizontal and 40 degrees vertical, so that hand movement was not visible. The experimental room was surrounded with black curtains, and the roof and floor were painted black.

**Figure 1:**
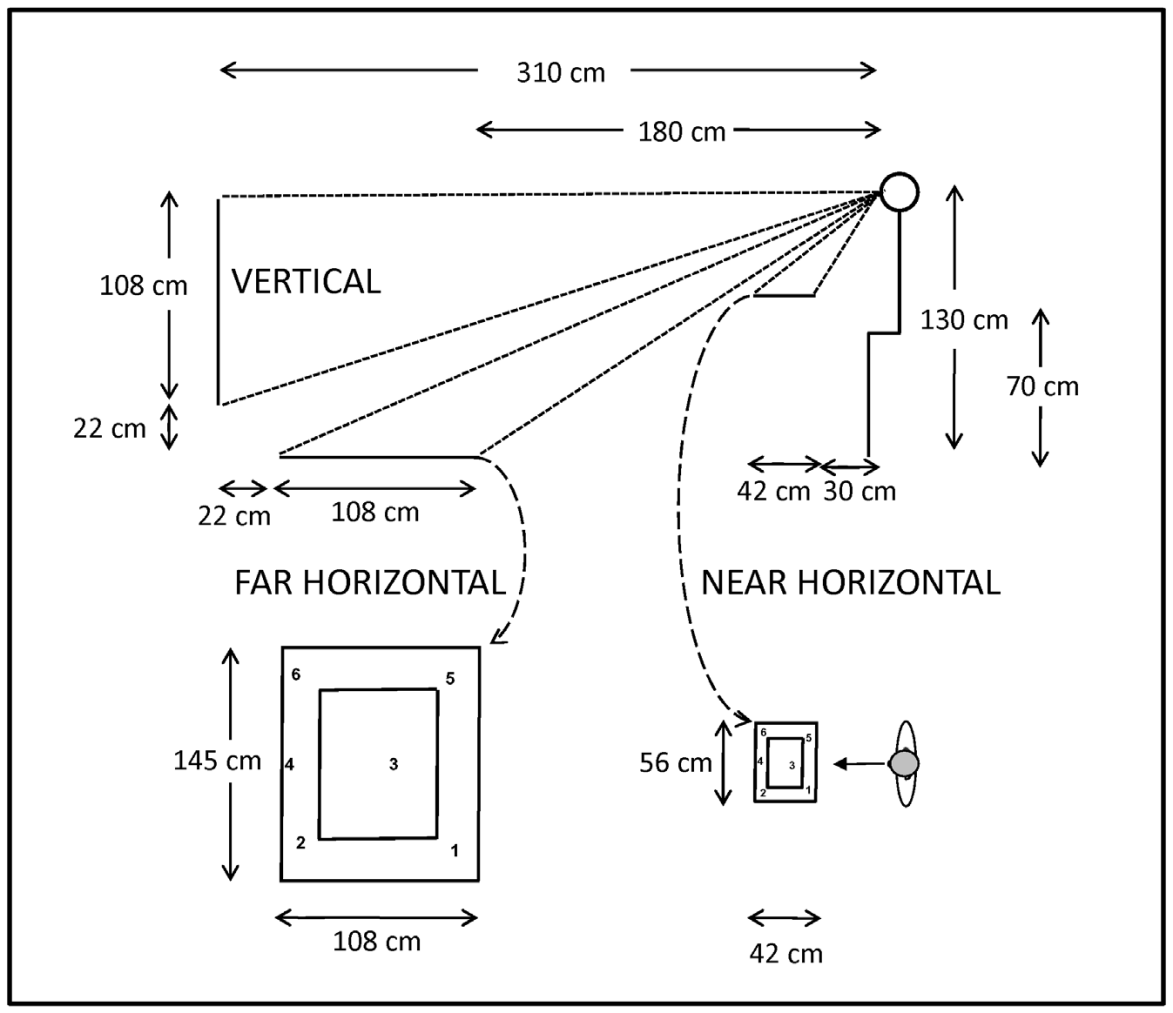
Schematic representation of the experimental setup for the pointing tasks: sagittal view (top) and top-view (bottom). The participant was seated in front of the three spaces on which red dots were projected as target stimuli. When the target disappeared, the participant moved the laser pointer to the memorized location. From the observer's position, the horizontal near and far spaces correspond to a viewing angle of approximately 40×24 degrees and 35×12 degrees in the horizontal plane, respectively. The vertical space corresponds to a viewing angle of approximately 26×19 degrees in the vertical plane. At bottom, the top-view shows the two concentric rectangles and the angular distribution of the targets in the near and far spaces. In the experiment, the rectangles are formed with white stripes on a black backdrop and are visible only in the light condition.

To distinguish the consequences of vestibular loss in both the peripersonal and extrapersonal spaces, the target location was assessed in two bi-dimensional horizontal spaces, the “near horizontal plane” and the “far horizontal plane” (Figure 1). The target location was evaluated in the left-right (contralesional-ipsilesional) and fore-aft (straight-ahead) directions. In addition, to clarify whether the potential error in the fore-aft direction might be associated with an error in the up-down (gravitational) direction, the target location was also analyzed in the vertical plane for five patients and six healthy participants.

The horizontal near space (56 cm wide, 42 cm deep, 70 cm high) corresponded to the gripping space. The horizontal far space (145 cm wide by 108 cm deep) was located on the ground 180 cm away from the participant. The vertical or fronto-parallel space (145 cm wide by 108 cm high) was located 310 cm in front of the participant and centered on the line of sight. A panel with red laser LEDs for the projection of target stimuli (0.6 cm diameter dots) was mounted perpendicular to each space and managed using a computer program (LabView v7.0, National Instruments, Austin, TX, USA).

The spatial arrangement of the targets was designed to prevent the participants from building a cognitive representation that could influence the accuracy of the pointing responses. Thus, the targets were located in directions and at distances that varied with respect to the participant's midline projection (Figure 1, bottom). However, in all spaces, two targets were displayed in the participant's left hemifield, two targets were displayed near the participant's midline (one target was aligned with the participant's midline) and two targets were displayed in the participant's right hemifield.

#### Procedure

The participants were required to point to a visual target that disappeared prior to movement. Each target was presented for 2 sec. When the target disappeared, the laser pointer was switched on and the participant was instructed to point to the memorized location immediately, as directly and as accurately as possible. No time constraint was imposed. After reaching the estimated target location, the participants were instructed to say “ok” and remain stationary. One experimenter used a fixed camera to take a snapshot of the response. Subsequently, the pointer was automatically switched off and moved to the starting location for the next trial.

The pointing tasks were performed in total darkness and in light using a structured background to provide visual references (two concentric rectangles formed with 0.8-cm-large white stripes on a black backdrop), without particular depth cues. The pointing tasks were performed in blocks of balanced ordered sessions. Three blocks were performed in the dark (one block for each space) and two blocks were performed in the light (for the horizontal near and far spaces). Each block was composed of 24 trials with four trials per target. The targets were randomly presented in each block, and no identical targets were presented in successive trials. A computer program displayed the targets one by one, and each response location was recorded for subsequent analysis. A five-minute resting period was provided between each block presentation. To become familiar with the tasks, the participants completed a few trials before starting data acquisition. The participants received no feedback during or after the experimental session.

#### Data collection and analysis

For calibration purposes, the target LED was activated and its location was automatically recorded before each session. To analyze the responses, each target location was compared with the participant's response for that target by computing the barycenter of the laser spot, applying the calibration corrections and computing the corresponding distances along the two axes. The barycenter computation was used to compensate for the unevenness of the target LED illumination spot and the small distortion of the spot resulting from the slight tremor of the participant's hands when holding the pointer. The resolution of the recording system was calculated considering the overall dimensions of the experimental field, the number of pixel rows and columns and the distance between the laser pointer and the experimental field. The resolution ranged from 0.1 to 0.2 degrees according to the target location.

The data (in millimeters) were transformed into degrees computed from the participants' eyes to obtain the direct comparison of the performance between the different spaces. Pointing to the right of the real target location (i.e. deviations to the ipsilesional side) was indicated with positive values, whereas negative values indicated a leftward pointing error (i.e. deviations to the contralesional side). The x- (left-right) and y- (fore-aft) errors were analyzed using a mixed-design analysis of Variance (ANOVA) with Group (UVD patients, controls) as the between-participant factor, and Session (n = 3), Condition (dark, light) and Space (far, near) as within-participant factors. For the representation of body pointing direction task (see below), mixed ANOVAs with Group and Session were performed. The significance level for all means was less than or equal to 0.05.

### Representation of body pointing direction

To determine whether the pointing error was associated with changes in representation of body pointing direction, the visual SSA was also recorded for each participant during each session. The SSA was assessed using a laser line (Framiral, Cannes, France) projected on the horizontal far plane and in the fore-aft direction with the rotation centered on the participant's midline. The participants were instructed to imagine the laser line starting from their navel and extending straight ahead from the trunk and to adjust the orientation of the pointer so that the two extremities were positioned on this virtual line. The experimenter placed the starting position of the line either to the left or to the right (two trials each) of the participant's midline projection. The participants were subsequently instructed to align the laser straight ahead using a pair of pushbuttons held in each hand. The orientation of the laser line relative to straight ahead was automatically measured using a motorized system. The mean SSA was expressed in degrees and calculated by averaging the four consecutive trials of each participant. For each patient, the positive and negative signs referred to ipsilesional and contralesional SSA deviations, respectively; for the healthy participants, the positive and negative signs referred to rightward and leftward deviations, respectively.

### Eye movements

The potential consequences of eye movements on the representation of the target location and SSA were evaluated. After unilateral vestibular loss, perceptual changes might be associated with a spontaneous nystagmus or an altered resting location of the eyes through vestibular imbalance. Spontaneous nystagmus induces a drift in visual perception. However, this disturbance has been primarily revealed in darkness and in the absence of visual fixation. In the present study, only a few patients reported a drift of the target for the last trials when fatigue was established. In these cases, the trials were eliminated.

Eye movements were recorded using videonystagmography. A CCD camera, illuminated with infrared LEDs, was fixed on the goggles in front of the non-dominant eye (left eye in all subjects but one healthy participant). Because eye movements were recorded while the participants monocularly viewed the targets, this task was performed after the pointing task. In both near and far spaces, the same targets as those of the pointing task were randomly presented one by one. The participants were required to fix each target. Eye movements were sampled at 25 Hz and recorded for 15 seconds. The data were processed offline using a method based on a mathematical dynamic neural network. Iris gray-level detection was used to obtain the iris print of each patient. To examine spontaneous nystagmus (in degrees), the slow phase velocity of horizontal, vertical and torsional components was measured when the participants gazed at a target. Eye movement processing has been described in detail by [34]. The ocular torsion of the patient was calculated as the difference (in degrees) between the eye torsional position after and before UVN. The iris signature of the patient was established from the preoperative recordings (D−1) and recognized automatically for further processing.

## Results

### Target location

#### Left-right pointing error in the horizontal plane

**Figure 2:**
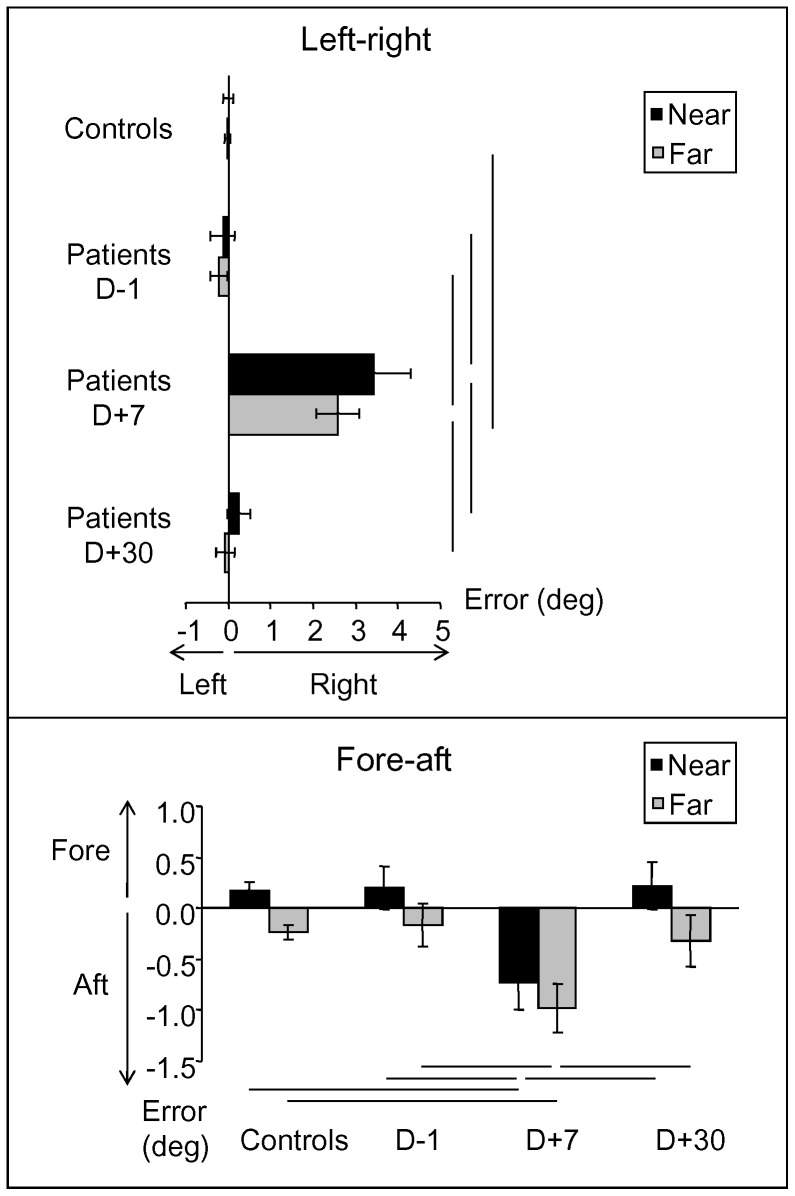
Effect of unilateral vestibular loss on pointing error in darkness. Average left-right (top) and fore-aft (bottom) pointing error for each group of participants in the near (black) and far (gray) spaces. Three sessions were plotted for the patients, while only the average values were plotted for the controls because the performances of these individuals remained consistent throughout the three sessions. Mean (±95% CI).

Significant effects of Group (F_1,24_ = 12.28; p<0.01) and Session (F_2,48_ = 20.15; p<0.001) were found for the pointing error in the left-right direction. The pointing error was significantly higher for patients than for healthy participants. A significant interaction between Group and Session (F_2,48_ = 18.08; p<0.001) showed that the pointing error differed over time more in patients than in healthy participants. Planned comparisons revealed that the effect of Session was obvious only for the patients (F_1,12_ = 12.26; p<0.01), indicating a drastic impairment in target location after unilateral vestibular loss. An overall effect of Space was also found (F_1,24_ = 11.17; p<0.01), with a higher pointing error observed in the near space than in the far space. A major effect of Condition (F_1,24_ = 14.83; p<0.001) and significant effects of the Group x Condition interaction (F_1,24_ = 14.88; p<0.001) and the Group x Space x Condition interaction (F_1,24_ = 4.68; p<0.05) were observed, indicating that the pointing error was differentially affected in patients and healthy participants as a function of space and condition (Table 2). To specify these data, planned comparisons were conducted separately on near and far spaces. Figure 2 (top) illustrates the pointing error in the left-right direction for the two groups of participants as a function of space in the dark. The pointing error significantly differed between patients and healthy participants for near (F_1,24_ = 13.12; p<0.01) and far (F_1,24_ = 9.62; p<0.01) spaces. The difference between the groups was obvious during the early stages following unilateral vestibular loss at D+7 for the near (p<0.001) and far (p<0.001) spaces; mean lateral deviations toward the operated side of 3.4±0.8° (Mean±CI) and 2.6±0.5° relative to the target location in the near and far spaces, respectively, were observed for patients, and deviations of 0.1±0.1° and 0.1±0.1° were observed in the healthy group. For patients, the pointing error regained preoperative values at D+30. Under light conditions, the mean pointing error was less than that observed in darkness. At D+7, only a slight difference was reported between patients (0.4±0.5°) and healthy participants (0.1±0.1°) for the near space (p<0.01).

**Table 2:** Average pointing error (degrees) for each target in the near and far spaces for each group of participants.

	Controls		Patients D−1		Patients D+7		Patients D+30	
	Near	Far	Near	Far	Near	Far	Near	Far
Target 1	1.1±0.3	0.2±0.2	0.3±1.0	0.2±0.6	5.3±3.0	3.4±1.4	1.3±0.8	0.4±0.7
Target 2	0.1±0.2	0.1±0.2	0.1±0.5	0.2±0.5	4.5±2.5	3.2±1.4	0.6±0.6	0.2±0.5
Target 3	0.1±0.2	0.1±0.1	−0.2±0.4	−0.2±0,4	2.8±1.8	2.6±1.5	0.2±0.4	−0.2±0.3
Target 4	0.1±0.2	0.1±0.2	−0.1±0.5	−0.2±0.5	3.0±1.6	2.4±1.1	0.3±0.3	−0.1±0.4
Target 5	−0.7±0.3	−0.4±0.2	−0.3±0.6	−0.4±0.6	3.4±1.4	2.2±1.3	−0.4±0.5	−0.2±0.2
Target 6	−0.5±0.3	−0.1±0.2	−0.7±0.8	−0.5±0.7	1.4±1.1	1.6±0.9	−0.5±0.5	−0.5±0.4

Three sessions were plotted for the patients, while only the average values were plotted for the controls because their variations across sessions were non-significant. Mean (±95% confidence intervals).

**Figure 3:**
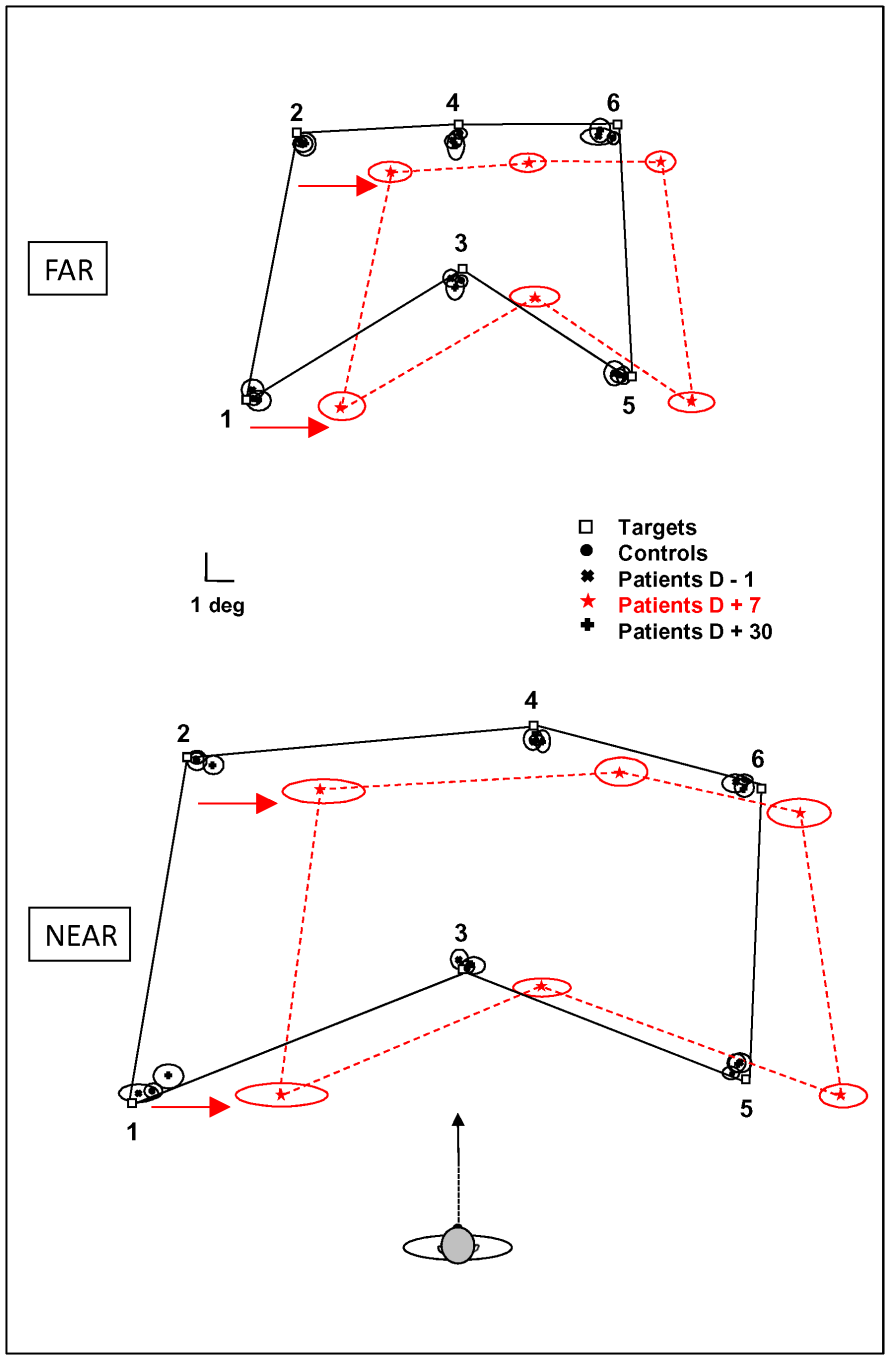
Pointing error in near and far spaces. Average pointing error (degrees) for each group of participants in the near (bottom) and far (top) spaces. The white squares correspond to the actual target locations. The distribution of targets and of pointing errors is presented to scale as seen from the observer's position, while the distances between the observer and the two spaces are not to scale. Three sessions were plotted for each patient: one day before UVN (D−1), one week after UVN (D+7), and one month after UVN (D+30). For clarity, only the average value was plotted in controls, as their variations across sessions were non-significant. The values were computed by averaging the x- (left-right direction) and y- (fore-aft distance) error of each group of participants. The 95% confidence intervals are also shown. In both spaces, the shift between the black full lines linking the target locations and the red dotted lines linking the maximal observed pointing error in patients at D+7 shows the systematic deviation of the memorized targets. A global space distortion toward the right side (highlighted by the red arrows) with a small underestimation of the distances was observed. As a result, the represented space is slightly compressed in the contralesional hemifield.

Interestingly, in the dark, the pointing error at D+7 depended on the location of the target respective to the patient's midline (Figure 3). For patients with right vestibular loss, the pointing error was consistently to the right side. For both spaces, the more targets that appeared to the right, the lower the pointing error was. In the near space, the pointing error was higher for left area targets (4.9±1.8°) than for both central (2.9±1.1°) and right area (2.4±0.9°) targets (p = 0.0005 and p = 0.004, respectively); the pointing error did not significantly differ for the central and right area targets. In the far space, the pointing error was higher for left area targets (3.3±0.9°) than for both central (2.5±0.9°) and right area (1.9±0.7°) targets (p = 0.006 and p = 0.0001, respectively), with a higher value detected for the central area than for the right area targets (p = 0.01). Therefore, considering the configuration formed by all targets, the represented space is distorted during the early stages following unilateral vestibular loss; this distortion is characterized by a shift toward the lesioned side and compression in the contralesional hemifield.

#### Fore-aft pointing error in the horizontal plane

In the fore-aft direction, the only factor affecting the mean pointing error was Space (F_1,24_ = 14.90; P<0.001); the pointing error was higher in the near space than in the far space. Significant effects of the Group x Session interaction (F_2,48_ = 5.08; p<0.01) and the Group x Condition x Session interaction (F_2,48_ = 9.57; p<0.001) were observed, suggesting changes in perceived target location after unilateral vestibular loss. These effects indicated that the pointing error was affected differentially over time for patients and healthy participants and only in darkness. Figure 2 (bottom) shows the pointing error in darkness for each space and session for patients and healthy participants. In darkness, the pointing error was higher for patients than for healthy participants, demonstrating an underestimation of the target location at D+7 in both far (patients: −1.0±0.24°; healthy participants: −0.3±0.1°; p<0.01) and near (patients: −0.7±0.3°; healthy participants: 0.2±0.1°; p<0.01) spaces. In the light, the performance of the patients and of the healthy participants was similar. In contrast to the pointing error in the left-right direction (revealing a distortion of the entire space), the pointing error in the fore-aft direction did not depend on the relative location of the target to the patient (Figure 3). Therefore, our data in the fore-aft direction show a global shift (underestimation) of the entire targeted area, without any difference by target laterality.

#### Up-down pointing and left-right error in the vertical plane

The pointing error was also measured in the vertical plane to clarify whether the error in the fore-aft direction in the horizontal plane (i.e. an error on the target distance estimate) is associated with an error in the up-down direction (i.e. an error on the target height estimate). For the fore-aft direction in the horizontal plane, an interaction between Group and Session (F_2,18_ = 0.43; p<0.01) was observed in the up-down direction. An additional ANOVA comparing the pointing error recorded in both planes revealed no significant differences between the up-down pointing error in the vertical plane and the fore-aft pointing error in the horizontal plane for both groups of participants. Moreover, an ANOVA was performed to assess whether the left-right pointing error in the vertical plane differed from that observed in the horizontal plane. The lack of significant differences revealed that the left-right pointing error was similar in both planes.

### Representation of body pointing direction

**Figure 4:**
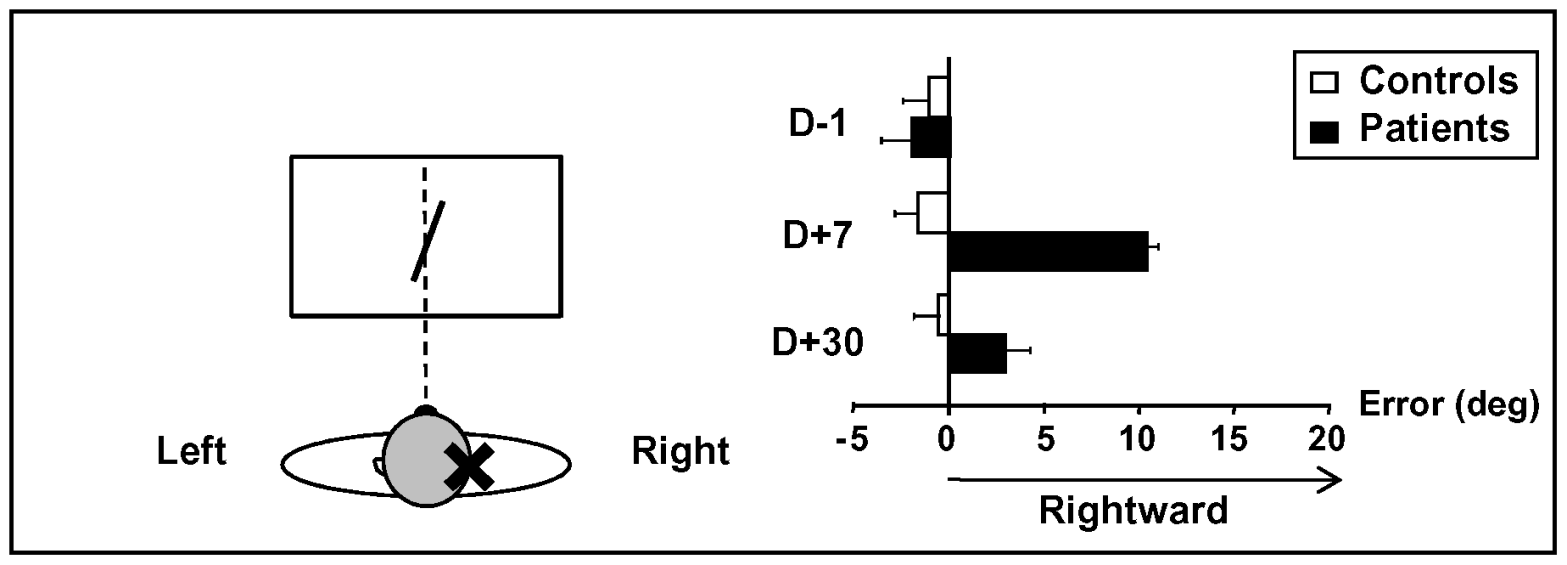
Effect of unilateral vestibular loss on the visual straight-ahead (SSA) as a function of session. Mean (±95% CI).

The variations in SSA for each session are illustrated in Figure 4. The mean SSA significantly differed between the two groups (F_1,24_ = 36.63; p<0.001) as a function of the experimental session (F_2,48_ = 20.61; p<0.001). A significant interaction between Group and Session (F_2,48_ = 25.01; p<0.001) revealed that SSA was differentially affected over time in the two groups of participants. The patients showed a rightward deviation (D+7: 10.6±2.5°, p<0.001 and D+30: 2.6±2.4°, p<0.05), while the healthy participants exhibited no such deviation (session 2: −1.3±1.1° and session 3: −0.4±1.4°). In addition, the patient SSA significantly decreased between D+7 and D+30 (p<0.001). During the first session, the directions indicated for the healthy participants and patients were close to the sagittal fore-aft orientation and did not significantly differ between the two groups.

### Eye movements

#### Spontaneous nystagmus and ocular torsion

A spontaneous nystagmus was observed in the dark and in the light during the early stages following unilateral vestibular loss (D+7), particularly for the horizontal component. Because participants were fixing a target, the spontaneous nystagmus velocity was weak (0.6±0.5°/s in darkness; 0.3±0.5°/s in light). In darkness, the pointing accuracy and spontaneous nystagmus responses were associated: both nystagmoid eye movements and spatial errors peaked at D+7, while no spontaneous nystagmus was observed at D−1 or at D+30, where there were no significant spatial errors. In addition, the data indicated that, for each visual condition, the velocity of the spontaneous nystagmus did not significantly differ according to target location. In darkness, the correlation between the velocity of spontaneous nystagmus and spatial error at D+7 for both spaces and each target location was not significant (Pearson r = 0.35, df = 11, p<0.05). No significant correlations were found when patients were looking either left (r = 0.41) or right (r = 0.45), in near space (r = 0.44) or in far space (r = 0.13). Finally, all patients displayed a static ocular torsion after UVN resulting from a rotation of the upper pole of both eyes toward the operated side. The mean ocular torsion was 7.5±1.6° at D+7 and 3.9±1.1° at D+30 toward the operated side.

## Discussion

This study assessed the representation of external space in patients with a complete unilateral vestibular loss. In the dark, the configuration of targets was shifted toward the lesioned side and compressed toward the contralesional hemifield. Patients underestimated the location of targets in both the fore-aft and up-down directions. Representation was impaired for both near and far spaces, while error was higher in the near space. In addition, this impairment was associated with an impairment of representation of body pointing direction.

### External space representation is impaired after unilateral vestibular loss

For the first time, data show that patients examined during the early stages following unilateral vestibular loss experience impairment in the spatial location of visual memorized targets in the dark, i.e., when participants rely on egocentric strategies [22]. The configuration of targets is shifted toward the lesioned side, i.e., the right side for the patient population. These results are consistent with those of healthy subjects showing that vestibular signals contribute to target location during self-motion [18]. Consistently, changes in vestibular signals caused by a modified gravito-inertial force field decrease the accuracy of spatial location of visual memorized targets [19]. Interestingly, impairments in target location reported in the present study occurred in the absence of subject motion, i.e., without vestibular stimulations other than those indicating that the head is stationary. In addition, the error depends on the target location relative to the patient's midline: the more displaced the targets are to the lesioned side, the lower the pointing error. Overall, the represented space is distorted and shifted toward the lesioned side, and the contralesional hemifield is compressed. We hypothesize that the asymmetric distortion of the represented space is due to modification of the central integration of sensory information. These data point to the need for interaction of different sensory cues (vestibular, proprioceptive, visual), providing redundant information for the elaboration of an accurate representation of the external space. In other words, the unilateral suppression of vestibular inputs produces a transient disorganization of the perceived location of objects at a high level of spatial processing. These data are in line with those of previous studies revealing an impairment of spatial memory after unilateral suppression of vestibular inputs [13], [14]. Electrophysiological recordings from single neurons and neuroimaging studies have proposed that target-related information in an egocentric reference frame and kinematic parameters of reaching are distributed throughout a large network that includes both frontal and parietal cortical areas (e.g., [20], [35], [36]). Interestingly, this network receives different types of sensory information, including vestibular projections, and is involved in spatial perception [15], [37]–[41].

Target location deviates toward the affected side for both near and far spaces, with a deviation that is higher for near space. This dissociation, together with the previously reported asymmetric distortion of the represented space, suggests that the disorders of the extrapersonal space representation are similar in patients with unilateral vestibular loss and in patients with spatial neglect [23], [24] (see review in [42]) or pseudoneglect [43]. This hypothesis is also based on anatomical substrates because common central areas, including the superior temporal cortex, the insula and the temporo-parietal junction, are both involved in the cortical vestibular network and frequently lesioned in spatial neglect [29], [44].

Pointing error is also reported in the fore-aft and in the up-down directions. In these cases, it might result from an underestimation of either the distance between the body and the target location or the target height. However, because errors in these two dimensions did not differ, our data do not distinguish distance and height errors in the target representation. The role of vestibular information has been previously reported for travelled distance representation in healthy subjects [45] and distance estimation in navigational tasks in patients with vestibular defects [13], [14]. Previous studies have also suggested an internal model of gravity to estimate target motion accessible through the visual system [40]. However, whether changes in vestibular information influence the representation of static objects in the up/down direction remains unclear. Finally, in contrast to the description for the medio-lateral space, no space distortion has been reported in either the fore-aft or up-down directions.

Targets are accurately located in a structured visual background, supplying references and leading participants to rely on allocentric strategies. Under these conditions, error is either much weaker than in the dark or completely absent. Similarly, the perceptual bias in the judgment of the visual target location resulting from ocular partial paralysis [46] or displacement (eye-press) (e.g., [47]) is abolished if the target is presented in a structured visual field. The reduction of the pointing error with visual background has also been observed after the loss of somatosensory modalities [48] and in healthy subjects [49], [50]. For patients with acute vestibular loss, a predominant role for allocentric cues reportedly improves the accuracy of SSA ([27], [28] and SVV [51], [52].

### Changes in egocentric reference frame

Since the representation of body pointing direction is involved in egocentric pointing, the effect of unilateral vestibular loss on SSA has also been investigated. In the present study, patients with unilateral vestibular loss show an ipsilesional rotation of the SSA in the horizontal plane. This is consistent with previous data reported acutely [27] or several years after unilateral vestibular loss [29]. These deviations suggest that SSA is a vestibular-dependent parameter of visual orientation. In addition, vestibular information contributes to egocentric heading direction estimation [53], [54]. The observed modification of the orientation of the egocentric reference frame through vestibular loss is also consistent with the hypothesis concerning the altered representation of the width of hemi-bodies initially proposed by Pierre Bonnier in 1905 in patients with peripheral vestibular disorders (see [26]) or brain damage [55], [56]. We propose that changes in the egocentric reference frame might contribute, at least partially, to the inaccurate location of targets in space. Indeed, the impairment of the target location is concomitant with a SSA deviation in the same direction. However, the location of an egocentric reference does not fully depend on representation of body pointing direction. Indeed, the error observed in the target location is smaller than the SSA deviation, and a lack of deviation in the target location is observed as early as one month after unilateral vestibular loss, while a residual disturbance of the SSA remains at the same post-operative time.

### Other potential influences

Considering the potential role of eye movements, we especially address the question about whether changes in external space representation are secondary (or perhaps causally related) to temporary nystagmoid eye movements and torsion or are simultaneous effects appearing in parallel. Data indicate that both nystagmoid eye movements and spatial errors peak at D+7, whereas no spontaneous nystagmus is observed at D−1 or at D+30, where there are no significant spatial errors. However, at D+7, the nystagmus velocity is not correlated with the extent of the spatial errors, either in the left versus right hemifields or in near versus far space. It should be noted that the nystagmus velocity is weak, which is probably due to the fact that patients were requested to fix a visual target. Dissociation between perceptive responses and eye movements has also been reported in similar patient populations, as the SSA deviation remains during the chronic stage following unilateral vestibular loss, when no patients have residual spontaneous nystagmus [29]. Regarding the impact of ocular torsion on both the SSA and the mean error of the target location, it appears that the amplitude differed, providing evidence against an exclusive functional link between the eye torsional position and the perceptive changes. In addition, the error in the representation of the target location was nearly suppressed when pointing was performed in the light with a structured background to supply visual references. However, it has been previously reported that the range of eye cyclotorsion after unilateral vestibular loss did not differ in the dark and in the light with visual references [51]. Therefore, the highly reduced or fully suppressed pointing error cannot reflect changes in the eye position. Taken together, these results suggest that even though the causal role of nystagmoid eye movements and torsion cannot be totally excluded, the arguments above highly suggest that spontaneous nystagmus and eye cyclotorsion are not the critical parameters involved in coding the representation of target location and of body pointing direction. Rather, changes in spatial representation are involved.

Finally, the fact that the deficits observed in the representation of external space and of body pointing direction may be partially associated with decreases in the attentional resources allocated to the task cannot be ignored (see [57]–[58] for extensive reviews, and [59]). Several indirect influences, including spontaneous nystagmus, deficient gaze stabilization during head movements and imbalance after unilateral vestibular loss, could have interfered with the tasks performed here. These influences are difficult to separate from direct vestibular influences, as these effects are components of the unilateral vestibular syndrome. Several authors have reported that maintaining equilibrium in challenging postures decreases performance in cognitive tasks in patients with vestibular defects [60], [61]. Similar conclusions have been reached from the analysis of the interactions between vestibulo-ocular processing and cognitive tasks [62]. However, in the present study it is unlikely that similar mechanisms interfered with the tasks, as participants were seated with their head immobilized.

## Conclusions

These results provide strong evidence that vestibular signals are involved in external space representation for spatial location of visual memorized targets in the dark, i.e., when participants are relying on an egocentric strategy. This evidence further supports the hypothesis that deviations in the representation of body pointing direction are partially responsible for the inaccurate location of targets and suggests that vestibular signals are necessary for the precise integration of sensory information to maintain accurate spatial representation. Consequently, the present study stresses the importance of the vestibular system in spatial cognition. Therefore, vestibular syndrome may also be considered a disorder of spatial representation and is thereby comparable to spatial neglect.
